# The Use of Genomic Screening for the Detection of Chromosomal Abnormalities in the Domestic Horse: Five New Cases of 65,XXY Syndrome in the Pura Raza Español Breed

**DOI:** 10.3390/ani14172560

**Published:** 2024-09-03

**Authors:** Mercedes Valera, Ayelén Karlau, Gabriel Anaya, Monika Bugno-Poniewierska, Antonio Molina, Ana Encina, Pedro J. Azor, Sebastián Demyda-Peyrás

**Affiliations:** 1 Departamento de Agronomía, Escuela Técnica Superior de Ingeniería Agronómica, Ctra. Utrera km 1, Universidad de Sevilla, 41013 Sevilla, Spain; mvalera@us.es (M.V.); mejoragenetica@lgancce.com (A.E.); 2 CONICET-Facultad de Ciencias Veterinarias, Universidad Nacional de La Plata, Calle 60 y 118 s/n, La Plata 1900, Argentina; akarlau@fcv.unlp.edu.ar; 3 Departamento de Genética, Universidad de Córdoba, 14071 Córdoba, Spain; ganayacalvo@uco.es (G.A.); ge1moala@uco.es (A.M.); 4 Department of Animal Reproduction, Anatomy and Genomics, Uniwersytet Rolniczy im, Hugona Kołłątaja w Krakowie, 31-120 Kraków, Poland; monika.bugno-poniewierska@urk.edu.pl; 5 Real Asociación Nacional de Criadores de Caballos de Pura Raza Española (ANCCE), 41012 Sevilla, Spain; pedroazor@lgancce.com

**Keywords:** CNA, cytogenetics, equine, genomic chromosomal screening, sex chromosomal abnormalities, 65,XXY

## Abstract

**Simple Summary:**

Sex chromosomal abnormalities are a major cause of reproductive failure in horses. While some horses with abnormal karyotypes exhibit reproductive abnormalities, others appear normal, remaining misdiagnosed until late age. The Pura Raza Español breeding program screens all horses for these abnormalities using genomics before adding them to the studbook. This process includes an initial assessment using the results of Short Tandem Repeat (STR) parentage testing, followed by confirmation using Single-Nucleotide Polymorphism (SNP) analysis for copy number aberrations. Hereby, we identified five new cases of 65,XXY syndrome among 27,330 foals over two breeding seasons. These horses were flagged as possibly affected by a chromosomal number aberration (CNA) due to abnormal STR and confirmed as 65,XXY by genomic analysis. All of them showed a male phenotype. One horse showed abnormal gonad development, whereas the others did not have visible abnormalities. This study represents the largest group of horses diagnosed with 65,XXY and emphasizes the importance of genomic screening for chromosomal testing.

**Abstract:**

Sex chromosomal abnormalities are a well-established cause of reproductive failure in domestic horses. Because of its difficult diagnosis, the Pura Raza Español breeding program established a routine screening for chromosomal abnormalities in all the horses prior to enrolling in the studbook. This genomic procedure combines an initial assessment based on the results from Short Tandem Repeat (STR) parentage testing followed by a Single-Nucleotide Polymorphism (SNP) based copy number aberration (CNA) confirmative analysis in positive cases. Using this methodology, we identified five new individuals carrying a 65,XXY chromosomal number aberration (CNA) among 27,330 foals enrolled over the past two reproductive seasons. The animals were initially flagged as CNA candidates due to abnormal results in STR testing. Subsequent analysis genotyping using an STR sex-linked dedicated panel and a medium-density SNP array in ECAX and ECAY confirmed the diagnosis as 65,XXY carriers. Four cases (upon sample availability) underwent further analysis using in situ fluorescent hybridization with ECAX and ECAY probes, showing identical results. Phenotypic analysis revealed abnormal gonad development in one of the cases, showing that the remaining four had a normal reproductive morphology. To our knowledge, this represents the largest number of horses exhibiting the equine form of Klinefelter syndrome (65,XXY) reported to date. Our study highlights the importance of genomic screening in the accurate detection of chromosomal abnormalities in horses.

## 1. Introduction

Chromosomal abnormalities associated with sex chromosomes have been a well-established cause of reproductive failure in domestic horses for more than 50 years [1,2]. This fact was clearly demonstrated by Power [3], who compiled data from 400 individuals carrying diverse types of chromosomal aberrations in the most comprehensive study performed to date. The author established that most stallions and mares carrying chromosomal abnormalities were infertile. Similar results were recently reported by Bugno-Poniewierska and Raudsepp [4], who presented 215 new cases in a 20-year longitudinal analysis. In both cases, the authors agree that ECAX monosomy (63,X) is the most prevalent CNA (copy number aberration) presentation in the species, followed by 64,XY DSD sex-reversal syndromes and cellular chimerisms. Phenotypically, several CNA syndromes were associated with abnormalities in the internal and external reproductive organs and infertility (such as the 64,XX, dsd, or whole-body chimerisms, among others [5]). These presentations are easily detectable by visual inspection, prompting cytogenetic testing for a final diagnosis [6]. However, most of the horses carrying CNA exhibit normal phenotypes until adulthood, when unexplained infertility becomes apparent [7]. These latter cases are much more difficult to detect, remaining undiagnosed, at least until the individual reaches reproductive age. 

Klinefelter syndrome is a well-documented chromosomal abnormality in humans, characterized by the inheritance of an extra X chromosome in the sex chromosome pair (47,XXY). This extra chromosome commonly undergoes inactivation in the early developmental stages, but in some cases, certain genes may escape (a phenomenon known as partial inactivation), becoming the cause of the physical abnormalities associated with the syndrome [8]. Klinefelter’s prevalence was set close to 1.5 cases among 1000 liveborn children and was phenotypically associated with infertility in adult life in 91–99% of the cases. Despite that horses showed overall rates of chromosomal abnormalities similar to the ones reported in humans [9], the inheritance of an extra X chromosome (a Klinefelter-like syndrome, 65,XXY) is a condition rarely reported. Twenty-five years ago, Makinen et al. [10] reported two cases of this syndrome: one involving a 65,XXY French Trotter stallion with normal sexual behavior (but small, soft testes, a small penis, and azoospermia) and the a Standardbred trotter with normal testes, azoospermia, and a mosaic karyotype of 64,XY/65,XXY. More recently Iannuzzi et al. [11] reported a new case of a sterile stallion with a normal phenotype that was azoospermic, and Kakoi et al. [12] reported a case in a Thoroughbred horse in Japan with no clear phenotype. These findings are consistent with the description provided by Bugno-Poniewierska and Raudsepp [4], wherein 65,XXY individuals exhibit a normal male phenotype and typical male behavior yet are commonly infertile due to azoospermia, with some cases also showing underdeveloped gonads. This lack of a clear phenotype underscores the importance of cytogenetic analysis in determining whether infertility could be due to a chromosomal abnormality. 

One of the major drawbacks of horse cytogenetics is the lack of laboratories able to perform comprehensive karyotyping analysis in horses worldwide [13]. For this reason, using karyotyping as a routine screening methodology for all potential breeders is not viable within breeding programs. This fact probably leads to a large number of individuals carrying CNA (copy number aberration) remaining undiagnosed. This is so that the Texas A&M Molecular Cytogenetics Laboratory (probably the largest and most advanced horse cytogenetic laboratory in the world) only reported ~220 cases of horses carrying CNA during a period of 20 years [4]. To overcome the lack of screening methodologies, Kakoi et al. [12] proposed the use of STR parentage test results to perform a quick screening of CNAs in 17,500 horses. Using a similar method, we detected several horses carrying CANs in the Pura Raza Español breed [7,14,15,16]. However, STR-based methodologies are not valid to detect, in a reliable manner, the presence of mosaicisms and complex karyotypes or refine the diagnosis among different CNA presentations. To improve this low diagnostic ability of STR-based methods, we recently developed and validated a methodology to detect CNA using the information provided by high-throughput SNP genotyping platforms [17]. This methodology, developed and validated in the Pura Raza Español breed, relies on the analysis of the log R ratio (LRR) and B allele frequency (BAF) of each SNP marker per individual, allowing for the detection of most of the aneuploidies described in horses in a reliable manner. Using a similar approach, Shilton et al. [18] detected CANs in early pregnancy losses in mares, and Nogueira et al. [19] detected the presence of a female-to-male (64,XX) pseudohermaphroditism in an Arabian foal. However, the use of this technology is still low in animal cytogenetics. 

The pseudoautosomal region (PAR) is a genomic region exhibiting a high degree of homology between the X and Y chromosomes in mammals [20]. In horses, PAR is located within the first 1.86 Mb of the *p* arm on ECAX [21] and in the distal part of the ECAY [22]. This genomic homology allows for the existence of heterozygous loci in ECAX in males. But also, PAR will display unique BAF and LRR patterns in the presence of CNA associated with the sex pair. Using these unique patterns, we successfully identified several chromosomal abnormalities in PRE, including 63,X and 63,X/64,XX mosaics. The same approach can be used to detect the presence of an extra chromosome, such as in the case of 65,XXY horses. However, to our knowledge, this is the first time that this approach has been employed to detect the presence of this type of CNA in horses.

The Pura Raza Español (PRE) horse (also known as the “Andalusian horse”) is one of the most recognized Spanish breeds worldwide. PRE is not only the most important horse breed in Spain but one of the most important worldwide, with more than 270,000 registered individuals in 65 different countries [23]. The PRE studbook is managed worldwide by the Real Asociación Nacional de Criadores de Caballos de Pura Raza Española (ANCCE) under a closed enrollment policy. Since 2021, the molecular genetics laboratory of the PRE-ANCCE integrated a mandatory chromosomal screening into the Pura Raza Español (PRE) breeding program as follows: all horses showing reproductive abnormalities in phenotypic evaluations or exhibiting abnormal or incongruent results in STR-based parentage tests are flagged as CNA candidates and derived to our veterinary genetic diagnosis and animal genomics laboratory (MERAGEM Group, University of Córdoba, Spain) for further evaluation using SNP genotyping. To date, approximately 30,000 individuals have already been included in this screening analysis, showing prevalences much lower than previously reported [24]. 

In this study, we report the detection and diagnosis of five individuals carrying a pure 65,XXY chromosomal complement within the PRE genomic screening program for chromosomal pathologies. We also demonstrated the importance and accuracy of the use of genomic methodologies to enhance the detection of chromosomal abnormalities in domestic horses. To our knowledge, this is the largest study reporting the existence of 65,XXY individuals in the species.

## 2. Materials and Methods

### 2.1. Animal Enrolment, Parentage Testing, and Cytogenetic Screening

In this study, we analyzed 27,330 individuals (49% males and 51% females) enrolled during two consecutive breeding seasons (2021/2022 and 2022/2023) within the Real Asociación Nacional de Criadores de Caballos de Pura Raza Española studbook [25]. All individuals were part of the breed’s molecular screening program for chromosomal abnormalities conducted during parentage testing. Blood samples were collected by ANCCE’s official veterinary services following the breeding association’s standardized protocol for registration in the studbook, which also includes a visual inspection of each animal by an official ANCCE veterinarian to determine phenotypic sex and assess morphological abnormalities. In all the cases, resampling and inspection were conducted within the first year of the individuals’ lives. 

Parentage verification was performed by the PRE-ANCCE molecular laboratory following the guidelines described by the International Association for Animal Genetics [26] for STR-based parentage in horses. In brief, DNA was first isolated from whole blood samples using commercial kits following the manufacturer’s protocols. Thereafter, allelic variants of 17 standardized STR markers were determined using the protocols described by Dimsoski [27]. In addition, a sex-associated dimorphic PCR fragment (amelogenin marker) was evaluated according to Hasegawa et al. [28] in the same reaction as a sex control. Those individuals showing an incompatibility between phenotypic and genetic sex or showing abnormal results in the parentage analysis were flagged as CNA candidates and further analyzed by complementary techniques. A detailed description of the STR marker genotyping procedure and protocols is available in Demyda-Peyras et al. [15].

### 2.2. Resampling of CNA Candidates

The individuals flagged as positive in the STR-based CNA screening were re-inspected by the ANCCE veterinarian services, including the collection of additional blood samples taken in EDTA (for genomic analysis) and Heparin-Na (for cell culture) vacuum-sealed collection tubes, as well as 50 hair follicles to obtain non-blood DNA isolations (aiming to rule out the presence of blood chimerism). Finally, a more careful and thorough phenotypic inspection, with a particular emphasis on the reproductive morphology, was also performed by the veterinarians. It is worth mentioning that one of the cases was only reanalyzed using blood and hair DNA isolations obtained from the PRE-ANCCE germplasm bank since the individual was located in a third country. Finally, to determine the existence of a possible inheritance pattern, DNA isolations from the progenitors of the individuals were provided by the ANCCE germplasm bank.

### 2.3. STR-Based Chromosomal Diagnosis

Samples were retested using a validated methodology for the detection of chromosomal abnormalities in the PRE breed according to Anaya et al. [29]. In brief, five STRs (*LEX003*, *UCDEQ502*, *TKY38*, *LEX026*, and *TKY270*) and one fragment (*AMEX*) located on ECAX and three fragments (*EcaYH12*, *AMEY*, and *SRY*) were amplified in a single multiplexed PCR reaction. Allelic variants were determined by capillary electrophoresis in the Biosystems 3130 xl DNA sequencer (Applied Biosystems, Foster City, CA, USA) at the Central Service for Research Support (SCAI) at the University of Córdoba, Spain. The raw data obtained were processed and genotyped with GeneMapper v5.0 (Applied Biosystems, Foster City, CA, USA). In all the cases, all the determinations were made using hair and blood DNA isolations separately to rule out the presence of blood chimerism.

### 2.4. SNP Genotyping and CNA Genomic Analysis

DNA isolations from the individuals and their parents were genotyped using the Illumina Equine GGP array V5 (71,778 SNPs, Neogen, Scotland, UK). Raw data (final report files) were transformed into CNV analysis files (including individual, marker name, chromosome and position, BAF, and LRR) using an in-house pipeline developed in R V4.4.1 [30]. In both cases, the initial dataset was pruned, retaining only the SNP located in the ECA5 (n = 2891), ECA15 (n = 2692), ECA25 (n = 1107, used as autosomal controls), ECAX (n = 3431), and ECAY (n = 172).

SNP-based CNA analysis was performed following the methodology described by Pirosanto et al. [17] with some modifications. In brief, we first clustered ECAX markers into two groups according to their positions as pseudoautosomal region markers (PAR) (located within the first 1.86 Mb of ECAX) and non-pseudoautosomal (NON-PAR) markers (located between 1.86 Mb and 128.21 Mb of ECAX). In each chromosome/region, we determined three different statistics per individual: the mean log R ratio value (LRR), % of heterozygosity (HET, estimated as the % of BAF markers with values > 0.25 and <0.75), and the *p*-value for the *dip test* for unimodality [31] performed on all the BAF values used for estimate HET. In all the cases, the calculations were performed in R using the *tidyverse* [32], *data.table* [33], and *diptest* [34] packages. Data visualization was performed using the *ggplot2* ecosystem in R 4.4.1 for Windows [35].

### 2.5. Cell Culture and Fluorescent In Situ Hybridization (FISH) Chromosomal Analysis

The chromosomal complements in the four individuals diagnosed as 65,XXY with cytogenetic samples available were also analyzed using in situ fluorescent hybridization (FISH) techniques. For this, we first performed chromosomal spreads from peripheral blood lymphocyte cultures following our standardized procedure. In brief, 10 mL fresh blood samples underwent centrifugation at 800× *g* for 10 min. Subsequently, the white cell interphase and 1 mL of autologous plasma were seed in 12 mL sterile tubes containing 8 mL of RPMI 1640 medium supplemented with 5 μg/mL pokeweed lectin, 100 IU penicillin/mL, 100 μg/mL streptomycin, and 250 ng/mL of amphotericin B (Sigma Aldrich, Madrid, Spain). Lymphocytes were cultured at 37 °C for 72 h in a horizontal position. Twice a day, the cultures were gently inverted 5 times manually to activate the cell proliferation. An hour before the harvest, 1 μg/mL colcemid was add to each tube. Thereafter, cell cultures were centrifuged (5 m at 800 g) and harvested, keeping only 1 mL at the bottom of the tube. In the next step, cells were submitted to a 25 m incubation in 0.075 M KCl hypotonic solution, recentrifuged, and harvested following the same procedure. Finally, the cells were double fixed in a cold methanol/acetic acid (3:1) solution and stored at −18 °C until analysis.

Metaphase spreads underwent analysis via fluorescent in situ hybridization (FISH) utilizing whole chromosome-painting probe-specific ECAY (Department of Animal Reproduction, Anatomy and Genomics, University of Agriculture in Krakow, Poland). This probe gives a specific signal on all Y chromosomes and an additional signal in the interstitial part of the long arm of the X chromosome (in the area with the heterochromatin band characteristic of this chromosome) and has been successfully used to analyze copies of both Y and X chromosomes [36]. The probe was generated by chromosome microdissection and DOP-PCR amplification and labeled for simultaneous double-color fluorescence hybridization following routine protocols [37]. A standard FISH protocol [38], with minor adjustments, was employed. The labeled probes were denatured at 70 °C for 10 min. Metaphase spreads were digested first with RNAse (10 mg/mL) at room temperature for 60 min and then with 0.02% pepsin solution at 37 °C for 10 min. After digestion, target metaphase spreads were denatured in a hybridization solution consisting of 30% 2× saline-sodium citrate (SSC) and 70% formamide (Sigma-Aldrich, Darmstadt, Germany) at 70 °C for 2.5 min. The probes were then applied onto the metaphase spreads, covered, sealed with rubber cement, and left to hybridize overnight in a dark, moist chamber at 37 °C. Post-hybridization washes comprised two rounds in 50% formamide in 2× SSC and two rounds in 1× SSC at 42 °C. Signals were detected and amplified using avidin-fluorescein isothiocyanate and anti-avidin antibodies (Sigma-Aldrich). Chromosome staining was carried out with 4′,6-diamidino-2-phenylindole (DAPI) (Cambio, Cambridge, UK). Microscopic analysis was performed under a fluorescence microscope (Axio Imager, Zeiss, Jena, Germany) equipped with Zeiss ZEN imager software 3.7. Spectrum Red filter was used to take the pictures.

### 2.6. Genetic Analysis and Inheritance Estimation

A genetic characterization was performed on the CNA candidates to determine the existence of an inheritance pattern or relativeness among the individuals showing the abnormal karyotype. To perform this, we determined the individual inbreeding (F) of each individual, which is defined as the probability that an individual has two identical alleles by descent [39], as estimated according to Meuwissen and Luo [40]. We also determined the coefficient of coancestry (*f_ij_*), which is defined as the probability that two gametes taken at random from two individuals belonging to the population have identical alleles [41]. Finally, we determined the ancestors explaining 50% of the variability of the reference population (*a_50_*), as well as the occurrence of common ancestors up to four generations. All the estimations were computed using ENDOG software v 4.8 [42] using an extended pedigree with all the information available related to the affected individuals, including 1714 animals.

## 3. Results

### 3.1. Detection of 65,XXY CNA by Molecular Screening

STR-based screening flagged five individuals (0.018% of the total population analyzed) as candidates for carrying sex-related CNA chromosomal complements (positive AMX and AMY and heterozygous call in the LEX33 STR marker located in ECAX). Four of the individuals were resampled and inspected by the ANCCE veterinary services for further analysis. DNA isolates from the hair and blood of the remaining individual (located outside Spain) were obtained from the ANCCE germplasm bank.

### 3.2. Animal Phenotyping

**Figure 1:**
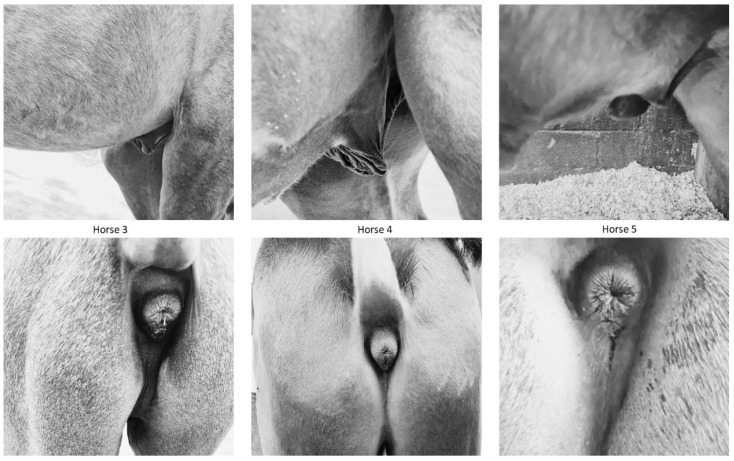
External reproductive tract of 3 of the 5 65,XXY horses analyzed in this study. In all the cases, morphology was normal with development according to their age.

All the individuals showed a male phenotype with a normal morphology, according to the age and the standard of the PRE breed. The reproductive tracts from Horses 2, 3, 4, and 5 were described as normal, with sexual development according to age. On the contrary, Horse 1 showed abnormal development of the gonads, presenting only a very small testicle detectable at palpation (monorquid). In all of the cases, the sperm quality of the individuals was not tested since they were still too young to collect sperm samples. Reproductive phenotypes of Horses 3, 4, and 5 can be seen in Figure 1.

### 3.3. Sex-Linked STR Chromosomal Analysis

The results of the sex-linked STR marker panel analysis are presented in Table 1. The five cases showed a high degree of heterozygosity in ECAX-linked STRs, ranging from two (UCDEQ502) to five (LEX003) polymorphic calls among the five individuals. This degree of allelic variability depicted the presence of two different copies of ECAX. In addition, the three ECAY-linked markers were amplified in all the samples, including the determination of the *SRY* gene, responsible for the male phenotype of the individuals. According to this analysis, the most probable chromosomal arrangements of the five animals was 65,XXY, but the presence of mosaic forms cannot be determined.

### 3.4. SNP-Based Chromosomal Analysis

The results of the SNP-based CNA analysis are presented in Table 2. 

All analyzed autosomes (ECA5, ECA15, and ECA25) exhibited increased heterozygosity (HET), ranging from 22.87% to 31.78%. Log R ratio (LRR) values were close to 0 (ranging from −0.06 to 0.08), and dip test *p*-values were near 1 (ranging from 0.79 to 1). The elevated HET and the LRR values close to 0 indicate the presence of heterozygous loci and a DNA abundance compatible with diploidy. Additionally, the non-significant results of the dip test suggest that the central region BAF histogram (BAF > 0.25 and BAF < 0.75) follows a unimodal distribution, which is consistent with the absence of trisomies since it does not exhibit the trimodal distribution associated with that kind of CNA (a detailed description of the procedure is available in Pirosanto et al. [17]).

In ECAY, four cases exhibited HET values close to zero and LRR values around −0.4 (ranging from −0.22 to −0.39). These results are indicative of a single copy of ECAY (HET close to 0 and LRR values near −0.5), associated with a hemizygous genome. However, Horse 1 showed an LRR close to 0, which may suggest the presence of two ECAY copies.

In the non-PAR region of ECAX, all individuals displayed a consistent pattern: HET~30%, LRR~0, and dip test *p*-values~1. This pattern suggests the presence of two chromosomal copies (increased HET indicates heterozygous loci, LRR values close to 0 indicate diploidy, and dip test values ruled out more than two chromosome copies) in all individuals.

**Figure 2:**
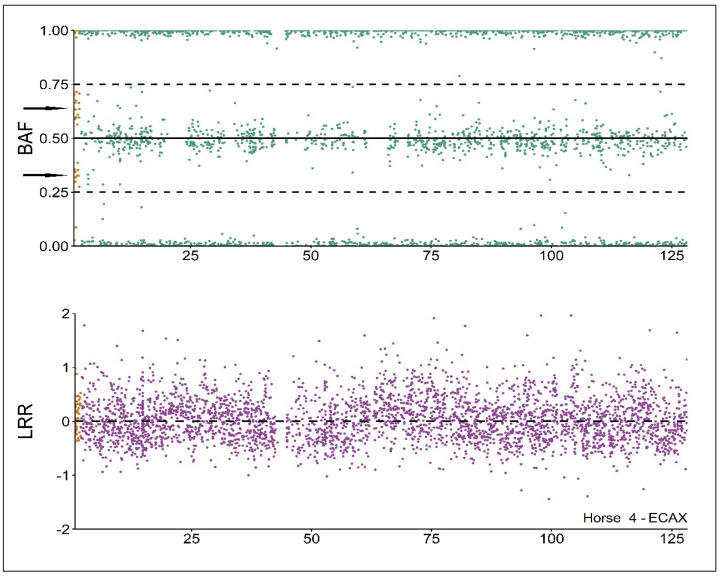
SNP−based CNA analysis results of Horse 4 ECAX. LRR is slightly higher in PAR (in yellow) than in NO−PAR (purple). BAF (upper part) shows a bimodal distribution in PAR (yellow part, two black arrows showing the modes), compatible with a trisomy and a unimodal distribution in NO-PAR (green).

Finally, The PAR region exhibited a consistent pattern across all individuals; heterozygosity (HET) was significantly higher than in other chromosomes, ranging between 40% and 58%, with an LRR of approximately 0.15 and a dip test *p*-value close to 0 (ranging between 0.04 and 0.25). The increased HET suggests the presence of more than two chromosomal copies in this region, which was confirmed by the characteristic chimeric-like bimodal BAF distribution observed (Figure 2). 

This BAF pattern was further supported by significantly lower dip test results compared to other normal diploid chromosomes, indicating a non-unimodal distribution. Together, these findings demonstrate the presence of three chromosomal copies (two ECAX and one ECAY). In addition, identical results were obtained from both blood and hair follicle DNA isolations in all five individuals, effectively ruling out the occurrence of blood chimerism. Additionally, analyses of the progenitors (five sires and five dams) showed normal chromosomal complements (64,XY and 64,XX, respectively), excluding the possibility of genetic inheritance of the CNA. Overall, these results confirm that the five individuals carried a non-inherited 65,XXY chromosomal complement.

### 3.5. In Situ Hybridization Analysis

**Figure 3:**
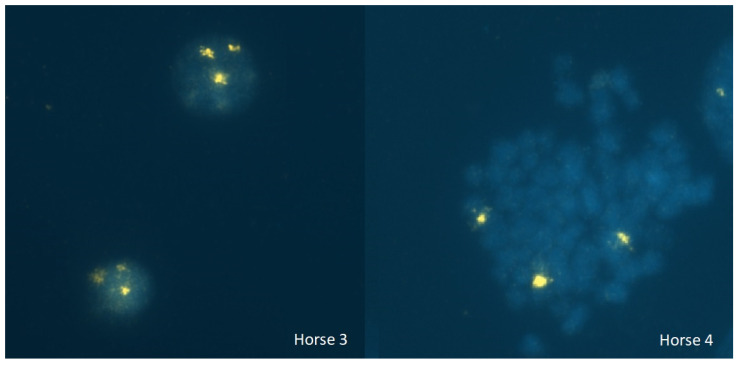
Fluorescent in situ hybridization chromosomal analysis. Interphase (Horse 3) and metaphase (Horse 4) FISH results. In both cases, the smaller and less brighten signals belong to ECAX, and the biggest and brighter belong to ECAY.

Four of the five individuals were also analyzed using FISH molecular karyotyping (it was not possible to collect cytogenetic samples from Horse 5 since it was sold to a third country outside Spain). All of them showed three different hybridization signals (two compatible with ECAX and one with ECAY) in all the cells analyzed (Figure 3), confirming the presence of a 65,XXY chromosomal complement. 

### 3.6. Genetic Analysis

The inbreeding coefficients (F) and coancestry coefficients (*f_ij_*) for the five animals carrying the 65,XXY karyotype are presented in Table 3. In all the cases, the inbreeding value was low, as well as the average coancestry among the individuals. No relationships among the five horses were detected in the four previous generations, ruling out a family inheritance of the genetic condition. The number of ancestors explaining 50% of the genetic variation among the five cases is low (11) but in agreement with the mean value obtained for a given individual of the PRE breed [43]. The number of foals per mare and the age of the mare at foaling the affected individual were also variable, suggesting that both effects were not related to the occurrence of the genetic condition in the foals.

## 4. Discussion

Chromosomal abnormalities are a common genetic issue in horses and show a much higher prevalence of these abnormalities compared to other livestock species [8]. However, the presence of XXY syndrome (a variation of the Klinefelter syndrome in humans) is extremely rare in horses. In this study, we present a two-step genomic approach, combining an STR-based screening with an SNP-based diagnostic test to detect the existence of horses carrying a 65,XXY chromosomal complement at a very young age. Using this innovative methodology, we report the largest number of individuals carrying a 65,XXY karyotype to date.

Two major drawbacks are associated with the lack of CNA detection in horses. First, the horse karyotype is complex, and only a few laboratories worldwide are capable of performing comprehensive cytogenetic analysis [4]. In addition, several sex-pair-related chromosomal syndromes are associated with normal phenotypes in young and adult individuals [13]. Therefore, it is common that individuals not intended for reproduction remain undiagnosed, thereby lowering the prevalence and knowledge existing in some specific CNAs (including 65,XXY) at the populational level. Our results estimated an incidence of this genetic condition close to 1 in 5500 in the PRE population and approximately 1 in 2600 in colts. The results agree with the only population-wide analysis evaluating the prevalence of 65,XXY in horses, which detected four cases in ~17,500 individuals [12]. These percentages are much lower than those reported in humans, where the prevalence is 1 in 500 individuals [44], but this is much higher than previous reports in most livestock species and horses, where the prevalence detected is close to 0 [9,45,46].

The use of parentage tests as a screening methodology to detect 65,XXY in horses has a methodological limitation that is worth mentioning. The parentage test STR panel employed includes only one ECAX-linked marker (LEX003), which shows a heterozygosity rate of ~0.75 in PRE horses [29]. In our case, the five individuals analyzed were heterozygous for LEX003, allowing its flagging as CNA candidates. However, since the existence of a 65,XXY chromosomal complement in an individual is flagged by showing heterozygous calls in LEX003 and positive amplification in ECAY markers, ~25% of the individuals carrying 65,XXY (1 in 17,500 approximately) may present results compatible with a normal male (homozygous LEX003 and positive ECAY markers), remaining undiagnosed. For this reason, we developed a complementary panel aiming to expand the number of ECAX-linked STR markers included to increase diagnostic accuracy, but it requires complementary testing of all the individuals to be effective. On the contrary, all these affected animals can be easily detected through SNP-based CNA analysis, which can be performed using the routinely generated data obtained from commercial SNP arrays [17]. 

In a very recent study, Bugno-Poniewierska et al. [9] reported overall CNA rates ranging from 0.5% to 4.7% in a population of 500 Polish horses. Most of these abnormalities were detected in mares, with no 65,XXY cases reported. Such high prevalences should be easily detected when large populations are analyzed (in our case, we should detect about ~500 individuals carrying CNAs). On the contrary, large populational screenings performed showed values 10 to 15 folds lower (ranging between 0.01 and 0.02% of prevalence upon the type of abnormality screened [11,13,23,45]). In this sense, it is feasible that small and autochthonous breeds, such as those analyzed by Bugno-Poniewierska et al. [9], may harbor increased percentages of CNAs due to specific genetic causes (breed effect). This possibility is further supported by similar rates reported by the same authors several years ago in the same breed [44]. Also, the detection of mosaic forms of ECAX-linked chromosomal abnormalities, which may escape the screening capability of STR-based methodologies, could partially explain these discrepancies. Similar results were also presented by Pienkowska-Schelling et al. [47], who demonstrated that mares carrying minor percentages of ECAX mosaicism were normal and fertile. Nonetheless, in both cases, the potential for abnormal results triggered by chromosomal abnormalities originating from the in vitro culture of the cells cannot be ruled out as an additional possible cause of elevated rates of low-level mosaicisms in studies involving karyotyping determinations. 

In this context, there are genomic methodologies capable of determining, to a certain degree, the existence of mosaicisms in chromosomal complements. For instance, Szczerbal et al. [48] identified the presence of CNAs using ddPCR, and our group demonstrated the viability of using SNP-based genotyping data for this purpose [17]. However, ddPCR requires a dedicated complementary test in each candidate horse, whereas SNP genotyping information can be obtained from genomic breeding programs on a routine basis. For instance, the PRE breed is transitioning from STR to SNP-based parentage tests, a shift expected to be completed in a few years. This transition involves the implementation of a dedicated SNP array, which includes specific markers to detect most chromosomal abnormalities described in horses reliably and automatically [49]. As a result, a more accurate estimation of CNA rates is anticipated within the breed in the coming years. But, this type of information will likely become globally accessible as the transition to SNP-based technologies continues across various horse breeds worldwide [50,51,52,53].

The use of genomic screening has significantly increased the detection of 65,XXY individuals in divergent breeds, such as Thoroughbreds and PREs (as observed in this study). However, the occurrence of non-mosaic XXY karyotype remains very rare in all domestic species, with only a few cases reported in cattle, river buffaloes, and horses [54]. Generally, individuals carrying XXY chromosomal complements are males with a normal phenotype, though some may exhibit gonadal dysgenesis or other reproductive alterations. For example, Iannuzzi et al. [11] evaluated a three-year-old Bardigiano trotter stallion that appeared chromosomally normal but was sterile. A FISH analysis confirmed that it was an XXY carrier. Similarly, Makinen et al. [10] studied a French Trotter stallion and an American Standardbred Trotter stallion clinically and cytologically using karyotyping. Both individuals exhibited normal libido and sexual behavior but faced fertility issues. In another case, Kubień et al. [55] examined a draft horse through phenotyping, karyotyping, and endocrine analysis. This individual was sterile, had small, soft, hypoplastic testes, and lacked a scrotum, which aligns with some of the phenotypes described in humans affected by Klinefelter syndrome [56]. In our study, only one individual showed an abnormal reproductive phenotype, whereas four of them were normal. Since all of them were normal in non-reproductive phenotypic aspects, the latter four cases were only flagged as candidates due to abnormal results in the genomic profile. Without this testing, these horses would not have been considered as candidates for CNA carriers, leading to an underestimation of the prevalence of this genetic condition in the species. In terms of fertility, only a preliminary breeding soundness evaluation was conducted on the individuals, resulting in one monorquid and four normal horses. However, sperm quality was not yet tested due to their young age, so their fertility remains unproven. Interestingly, the lack of sperm quality is a phenotypic trait widely associated with previous cases of horses carrying 65,XXY and humans carrying 47,XXY karyotypes. Therefore, the occurrence of individuals with a lack of sperm quality could be a more than justified cause to perform a chromosomal test [13].

Pedigree-based analysis suggests that inbreeding, average relatedness, age of the mare, and/or the existence of a commonly affected ancestor have not triggered the occurrence of this CNA in the individuals analyzed. The inbreeding values and average coancestry levels in the affected individuals are relatively low compared to the average values observed in the PRE [57]. Similarly, the average number of ancestors explaining 50% of the genetic variability of each individual (~11) is close to the value observed in the entire PRE population (~10, according to Poyato-Bonilla et al. [43]). These results, combined with the absence of common ancestors within four generations and the normal SNP-based CNA testing performed in the parents of the affected cases, rule out the existence of an inherited genetic condition, as is described in humans [56]. However, it is noteworthy that this lack of inheritance of numerical chromosomal abnormalities is probably caused by the infertility commonly associated with 65,XXY carries.

The inbreeding analysis of four of the mares showed relatively low coefficients, except for the dam of Horse 1, which exhibited very high values (18.3%). Interestingly, this individual was the only one displaying an abnormal reproductive phenotype. However, a single case does not allow for the establishment of a causative association between these situations in this study. Finally, while chromosomal abnormalities in humans, including the occurrence of Klinefelter syndrome, are highly associated with maternal age [58,59], the age of the dam and the number of offspring produced by the mare were highly variable among the five cases, suggesting that these factors were not significantly associated with the trigger of this genetic condition.

## 5. Conclusions

This study represents the largest populational analysis of horses carrying a 65,XXY karyotype reported to date. We presented five cases of horses carrying such chromosomal syndrome, diagnosed using a combined cytogenetic and genomic approach. The prevalence of the syndrome was 0.018% in a large population of ~27,000 horses analyzed. Four of the animals were phenotypically normal, whereas one of them was monorquid. However, fertility has not yet been tested. Finally, our results show that genomic methodologies are valuable for improving the detection of chromosomal abnormalities in horses, highlighting the importance of cytogenetic screening and enhancing the detection of chromosomal abnormalities within breeding programs.

## Figures and Tables

**Table 1:** Results of the STR-based chromosomal analysis.

		Horse 1	Horse 2	Horse 3	Horse 4	Horse 5
ECAX markers	TKY38	105/129	129	111/129	107/129	105/129
	UCDEQ502	164	164/174	164	116/174	162/164
	LEX003	194/210	214/216	206/210	208/210	212/214
	LEX026	312/314	316	308	314	300/314
	TKY270	168	168/170	168/172	170/172	170/172
	AMEX	+	+	+	+	+
ECAY markers	AMEY	+	+	+	+	+
	EcaYH12	+	+	+	+	+
	SRY	+	+	+	+	+

ECAX-linked STRs are expressed as allelic length (in bp). AMEX and ECAY markers are expressed as presence (+).

**Table 2:** Results of the SNP-based CNA analysis.

	ECA	5	15	25	PAR	NO-PAR	Y
Horse 1	HET (%)	31.61	31.78	29.59	40.00	32.83	0.00
	LRR	0.04	0.08	0.04	0.14	0.03	−0.05
	BAF dip test	0.95	0.99	0.99	0.25	0.99	1
Horse 2	HET (%)	24.95	30.87	26.92	63.89	26.47	4.13
	LRR	0.03	0.06	−0.01	0.10	0.07	−0.22
	BAF dip test	1	1	0.89	0.04	0.93	0.84
Horse 3	HET (%)	22.87	25.19	27.55	50.00	29.19	3.25
	LRR	0.02	0.02	−0.03	0.13	0.04	−0.16
	BAF dip test	0.97	0.99	0.97	0.11	1	0.92
Horse 4	HET (%)	31.87	32.95	29.99	58.33	28.63	2.44
	LRR	0.04	0.06	0.03	0.12	0.03	−0.38
	BAF dip test	0.96	0.98	1	0	1	1
Horse 5	HET (%)	30.87	29.87	23.04	52.78	32.56	2.44
	LRR	0.02	0.04	0.01	0.12	0.03	−0.39
	BAF dip test	0.98	0.94	0.79	0.09	0.99	1

HET, LRR, and BAF values were obtained directly from the raw data. Dip test analysis on BAF data was expressed as a *p*-value. The results are clustered in three autosomes (ECA5, ECA15, and ECA25), two ECAX regions (PAR and NO-PAR), and ECAY.

**Table 3:** Populational genetic characterization of the individuals carrying CNA.

Id	Born	*F*	*F* of the Mare	Foals per Mare	Age of the Mare	Average *f_ij_*
Horse 1	27 April 2021	5.13%	18.89%	2	8	5.19%
Horse 2	3 March 2022	5.86%	6.37%	3	9	5.91%
Horse 3	12 December 2022	5.58%	5.03%	6	10	5.75%
Horse 4	28 March 2023	7.59%	4.56%	8	16	5.06%
Horse 5	15 July 2023	4.54%	5.92%	1	9	5.09%

*F*: inbreeding coefficient; *f_ij_*: coancestry coefficient.

## Data Availability

STR parentage data and biological samples employed in this study are owned by the ANCCE studbook and remain confidential. SNP genotyping data were produced, analyzed, and managed under a collaboration agreement between the ANCCE and MERAGEM groups. Since they involve individuals belonging to commercial herds, they can be only available for scientific purposes upon reasonable request to the authors.

## References

[1] Payne H.W., Ellsworth K., DeGroot A. (1968). Aneuploidy in an infertile mare. J. Am. Vet. Med. Assoc..

[2] Basrur P.K., Kanagawa H., Gilman J.P. (1969). An equine intersex with unilateral gonadal agenesis. Can. J. Comp. Med..

[3] Power M.M. (1990). Chromosomes of the horse. Domestic Animal Cytogenetics.

[4] Bugno-Poniewierska M., Raudsepp T. (2021). Horse Clinical Cytogenetics: Recurrent Themes and Novel Findings. Animals.

[5] Lear T.L., McGee R.B. (2012). Disorders of sexual development in the domestic horse, Equus caballus. Sex. Dev..

[6] Albarella S., De Lorenzi L., Catone G., Magi G.E., Petrucci L., Vullo C., D’Anza E., Parma P., Raudsepp T., Ciotola F. (2018). Diagnosis of XX/XY Blood Cell Chimerism at a Low Percentage in Horses. J. Equine Vet. Sci..

[7] Anaya G., Moreno-Millan M., Bugno-Poniewierska M., Pawlina K., Membrillo A., Molina A., Demyda-Peyras S. (2014). Sex reversal syndrome in the horse: Four new cases of feminization in individuals carrying a 64,XY SRY negative chromosomal complement. Anim. Reprod. Sci..

[8] Groth K.A., Skakkebæk A., Høst C., Gravholt C.H., Bojesen A. (2013). Klinefelter syndrome—A clinical update. J. Clin. Endocrinol. Metab..

[9] Bugno-Poniewierska M., Jankowska M., Raudsepp T., Kowalska K., Pawlina-Tyszko K., Szmatola T. (2024). Molecular cytogenetic screening of sex chromosome abnormalities in young horse populations. Equine Vet. J..

[10] Makinen A., Katila T., Andersson M., Gustavsson I. (2000). Two sterile stallions with XXY-syndrome. Equine Vet. J..

[11] Iannuzzi L., Di Meo G.P., Perucatti A., Spadetta M., Incarnato D., Parma P., Iannuzzi A., Ciotola F., Peretti V., Perrotta G. (2004). Clinical, cytogenetic and molecular studies on sterile stallion and mare affected by XXY and sex reversal syndromes, respectively. Caryologia.

[12] Kakoi H., Hirota K., Gawahara H., Kurosawa M., Kuwajima M. (2005). Genetic diagnosis of sex chromosome aberrations in horses based on parentage test by microsatellite DNA and analysis of X- and Y-linked markers. Equine Vet. J..

[13] Laseca N., Anaya G., Pena Z., Pirosanto Y., Molina A., Demyda Peyras S. (2021). Impaired Reproductive Function in Equines: From Genetics to Genomics. Animals.

[14] Anaya G., Fernandez M.E., Valera M., Molina A., Azcona F., Azor P., Sole M., Moreno-Millan M., Demyda-Peyras S. (2018). Prevalence of twin foaling and blood chimaerism in purebred Spanish horses. Vet. J..

[15] Demyda-Peyras S., Membrillo A., Bugno-Poniewierska M., Pawlina K., Anaya G., Moreno-Millan M. (2013). The use of molecular and cytogenetic methods as a valuable tool in the detection of chromosomal abnormalities in horses: A case of sex chromosome chimerism in a Spanish purebred colt. Cytogenet. Genome Res..

[16] Demyda-Peyras S., Anaya G., Bugno-Poniewierska M., Pawlina K., Membrillo A., Valera M., Moreno-Millan M. (2014). The use of a novel combination of diagnostic molecular and cytogenetic approaches in horses with sexual karyotype abnormalities: A rare case with an abnormal cellular chimerism. Theriogenology.

[17] Pirosanto Y., Laseca N., Valera M., Molina A., Moreno-Millan M., Bugno-Poniewierska M., Ross P., Azor P., Demyda-Peyras S. (2021). Screening and detection of chromosomal copy number alterations in the domestic horse using SNP-array genotyping data. Anim. Genet..

[18] Shilton C.A., Kahler A., Davis B.W., Crabtree J.R., Crowhurst J., McGladdery A.J., Wathes D.C., Raudsepp T., de Mestre A.M. (2020). Whole genome analysis reveals aneuploidies in early pregnancy loss in the horse. Sci. Rep..

[19] Nogueira P.P.O., Amorim G., Oliveira O.M., Demyda-Peyras S., Santos B.M., Mota L. (2021). Sex Reversal Syndrome in an Egyptian Arabian Horse Detected Using Genomic Data—A case report. J. Equine Vet. Sci..

[20] Raudsepp T., Das P.J., Avila F., Chowdhary B.P. (2012). The Pseudoautosomal Region and Sex Chromosome Aneuploidies in Domestic Species. Sex. Dev..

[21] Raudsepp T., Chowdhary B.P. (2008). The horse pseudoautosomal region (PAR): Characterization and comparison with the human, chimp and mouse PARs. Cytogenet. Genome Res..

[22] Janecka J.E., Davis B.W., Ghosh S., Paria N., Das P.J., Orlando L., Schubert M., Nielsen M.K., Stout T.A.E., Brashear W. (2018). Horse Y chromosome assembly displays unique evolutionary features and putative stallion fertility genes. Nat. Commun..

[23] Solé M., Valera M., Fernández J. (2018). Genetic structure and connectivity analysis in a large domestic livestock meta-population: The case of the Pura Raza Español horses. J. Anim. Breed. Gen..

[24] Demyda-Peyras S., Laseca N., Anaya G., Kij-Mitka B., Molina A., Karlau A., Valera M. (2023). Prevalence of Sex-Related Chromosomal Abnormalities in a Large Cohort of Spanish Purebred Horses. Animals.

[25] ANCCE Pura Raza Español Horse Breed Studbook. https://www.lgancce.com/web/.

[26] ISAG Equine Genetics and Thoroughbred Parentage Testing Guideline. https://www.isag.us/committee_members.asp?comm=IS-EGTP.

[27] Dimsoski P. (2003). Development of a 17-plex microsatellite polymerase chain reaction kit for genotyping horses. Croat. Med. J..

[28] Hasegawa T., Sato F., Ishida N., Fukushima Y., Mukoyama H. (2000). Sex determination by simultaneous amplification of equine SRY and amelogenin genes. J. Vet. Med. Sci..

[29] Anaya G., Molina A., Valera M., Moreno-Millan M., Azor P., Peral-Garcia P., Demyda-Peyras S. (2017). Sex chromosomal abnormalities associated with equine infertility: Validation of a simple molecular screening tool in the Purebred Spanish Horse. Anim. Genet..

[30] R-Core-Team (2024). A Language and Environment for Statistical Computing.

[31] Hartigan J.A., Hartigan P.M. (1985). The Dip Test of Unimodality. Ann. Stat..

[32] Wickham H., Averick M., Bryan J., Chang W., McGowan L., François R., Grolemund G., Hayes A., Henry L., Hester J. (2019). Welcome to the Tidyverse. J. Open Sourc. Softw..

[33] Dowle M., Srinivasan A. (2019). Data.Table: Extension of Data.Frame, R Package Version 1.12. 8. Manual. https://www.rdocumentation.org/packages/data.table/versions/1.12.8.

[34] Maechler M. (2024). Diptest: Hartigan’s Dip Test Statistic for Unimodality R Package, v0.77. https://cran.r-project.org/web/packages/diptest/index.html.

[35] Wickham H. (2016). Ggplot2: Elegant Graphics for Data Analysis.

[36] Pieńkowska-Schelling A., Bugno M., Owczarek-Lipska M., Schelling C., Słota E. (2006). Probe generated by Y chromosome microdissection is useful for analysing the sex chromosomes of the domestic horse. J. Anim. Feed Sci..

[37] Bugno M., Slota E., Pienkowska-Schelling A., Schelling C. (2009). Identification of chromosome abnormalities in the horse using a panel of chromosome-specific painting probes generated by microdissection. Acta Vet. Hung..

[38] Pinkel D., Straume T., Gray J.W. (1986). Cytogenetic analysis using quantitative, high-sensitivity, fluorescence hybridization. Proc. Natl. Acad. Sci. USA.

[39] Wright S. (1931). Evolution in Mendelian Populations. Genetics.

[40] Meuwissen T.H.E., Luo Z. (1992). Computing Inbreeding Coefficients in Large Populations. Genet. Sel. Evol..

[41] Falconer D., MCKay T. (1996). Introduction to Quantitative Genetics.

[42] Gutierrez J.P., Goyache F. (2005). A note on ENDOG: A computer program for analysing pedigree information. J. Anim. Breed. Genet..

[43] Poyato-Bonilla J., Laseca N., Demyda-Peyras S., Molina A., Valera M. (2022). 500 years of breeding in the Carthusian Strain of Pura Raza Espanol horse: An evolutional analysis using genealogical and genomic data. J. Anim. Breed. Genet..

[44] NHI How Many People Are Affected by or at Risk for Klinefelter Syndrome (KS)?. https://www.nichd.nih.gov/health/topics/klinefelter/conditioninfo/risk.

[45] Bugno M., Slota E., Koscielny M. (2007). Karyotype evaluation among young horse populations in Poland. Schweiz. Arch. Tierheilkd..

[46] Ducos A., Revay T., Kovacs A., Hidas A., Pinton A., Bonnet-Garnier A., Molteni L., Slota E., Switonski M., Arruga M.V. (2008). Cytogenetic screening of livestock populations in Europe: An overview. Cytogenet. Genome Res..

[47] Pienkowska-Schelling A., Kaul A., Schelling C. (2020). X chromosome aneuploidy and micronuclei in fertile mares. Theriogenology.

[48] Szczerbal I., Nowacka-Woszuk J., Kopp-Kuhlman C., Mackowski M., Switonski M. (2020). Application of droplet digital PCR in diagnosing of X monosomy in mares. Equine Vet. J..

[49] Encina A., Valera M., Laseca N., Rodrigues A., Demyda Peyrás S., Perdomo-González D., Azor-Ortiz P.J., Gil A., Ripolles M., Anaya G. Design of a Medium-Density chip for the genetic management of the Pura Raza Español and related breeds. Proceedings of the International Committee for Animal Recording Conferece.

[50] Aminou O., Badaoui B., Machmoum M., Piro M. (2024). Evaluation of the effectiveness of single nucleotide polymorphisms compared to microsatellite markers for parentage verification in Moroccan horses. Anim. Genet..

[51] Ishige T., Kikuchi M., Kakoi H., Hirota K.I., Ohnuma A., Tozaki T., Hirosawa Y., Tanaka S., Nagata S.I. (2023). Evaluation of parentage testing using single nucleotide polymorphism markers for draft horses in Japan. Anim. Sci. J..

[52] Lee S.Y., Kim S.M., Oyungerel B., Cho G.J. (2024). Single nucleotide polymorphisms for parentage testing of horse breeds in Korea. Anim. Biosci..

[53] Nolte W., Alkhoder H., Wobbe M., Stock K.F., Kalm E., Vosgerau S., Krattenmacher N., Thaller G., Tetens J., Kühn C. (2022). Replacement of microsatellite markers by imputed medium-density SNP arrays for parentage control in German warmblood horses. J. Appl. Genet..

[54] Villagómez D.A.F., Parma P., Radi O., Di Meo G., Pinton A., Iannuzzi L., King W.A. (2009). Classical and molecular cytogenetics of disorders of sex development in domestic animals. Cytogenet. Genome Res..

[55] Kubień E.M., Pozor M.A., Tischner M. (1993). Clinical, cytogenetic and endocrine evaluation of a horse with a 65,XXY karyotype. Equine Vet. J..

[56] Tüttelmann F., Gromoll J. (2010). Novel genetic aspects of Klinefelter’s syndrome. Mol. Hum. Reprod..

[57] Perdomo-Gonzalez D.I., Laseca N., Demyda-Peyras S., Valera M., Cervantes I., Molina A. (2022). Fine-tuning genomic and pedigree inbreeding rates in equine population with a deep and reliable stud book: The case of the Pura Raza Espanola horse. J. Anim. Sci. Biotechnol..

[58] Nagaoka S.I., Hassold T.J., Hunt P.A. (2012). Human aneuploidy: Mechanisms and new insights into an age-old problem. Nat. Rev. Gen..

[59] Bojesen A., Juul S., Gravholt C.H. (2003). Prenatal and postnatal prevalence of Klinefelter syndrome: A national registry study. J. Clin. Endocrinol. Metab..

